# CAL-Tutor: A HoloLens 2 Application for Training in Obstetric Sonography and User Motion Data Recording

**DOI:** 10.3390/jimaging9010006

**Published:** 2022-12-29

**Authors:** Manuel Birlo, Philip J. Eddie Edwards, Soojeong Yoo, Brian Dromey, Francisco Vasconcelos, Matthew J. Clarkson, Danail Stoyanov

**Affiliations:** 1 Wellcome/EPSRC Centre for Interventional and Surgical Sciences (WEISS), Charles Bell House, 43–45 Foley Street, London W1W 7TY, UK; 2 UCL Interaction Centre (UCLIC), University College London, 66-72 Gower Street, London WC1E 6EA, UK; 3 UCL EGA Institute for Women’s Health, Medical School Building, 74 Huntley Street, London WC1E 6AU, UK

**Keywords:** augmented reality, ultrasound training, HoloLens 2

## Abstract

Obstetric ultrasound (US) training teaches the relationship between foetal anatomy and the viewed US slice to enable navigation to standardised anatomical planes (head, abdomen and femur) where diagnostic measurements are taken. This process is difficult to learn, and results in considerable inter-operator variability. We propose the CAL-Tutor system for US training based on a US scanner and phantom, where a model of both the baby and the US slice are displayed to the trainee in its physical location using the HoloLens 2. The intention is that AR guidance will shorten the learning curve for US trainees and improve spatial awareness. In addition to the AR guidance, we also record many data streams to assess user motion and the learning process. The HoloLens 2 provides eye gaze, head and hand position, ARToolkit and NDI Aurora tracking gives the US probe positions and an external camera records the overall scene. These data can provide a rich source for further analysis, such as distinguishing expert from novice motion. We have demonstrated the system in a sample of engineers. Feedback suggests that the system helps novice users navigate the US probe to the standard plane. The data capture is successful and initial data visualisations show that meaningful information about user behaviour can be captured. Initial feedback is encouraging and shows improved user assessment where AR guidance is provided.

## 1. Introduction

Ultrasound is a vital tool in obstetrics, but it can be difficult to achieve consistent training that leads to good diagnostic performance [1]. The operator needs to appreciate the US appearance of foetal anatomy in order to build a mental three-dimensional (3D) model of foetal location. The accurate acquisition of image planes is vital to achieve consistent biometric measurements [2]. Results vary depending on operator experience and anatomical plane navigation presents a significant challenge to the inexperienced trainee [3]. Standard measurements such as the head circumference (HC), abdomen circumference (AC) and femur length (FL) provide diagnostic information on foetal development. Consistency in these measurements is not guaranteed, however, especially for less experienced US trainees.

To address some of these challenges in ultrasound training, we present a HoloLens 2-based mixed reality (MR) application named ‘CAL-Tutor’ that assists the trainee by providing two holographic visualisations:

- A view of both the foetal anatomy and the ultrasound slice in their correct physical location;
- Mixed reality guidance during US probe navigation to the three standard planes—HC, AC and FL.

**Figure 1:**
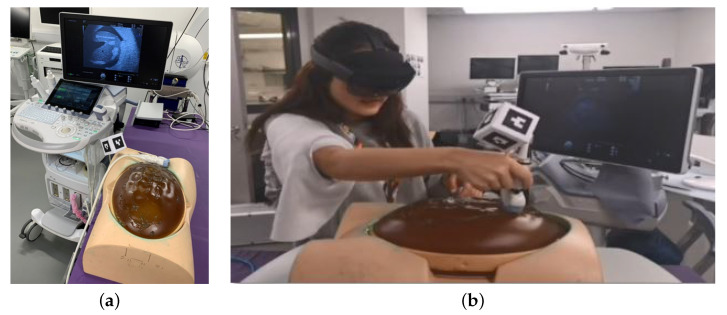
System design, showing (**a**) the setup of the ultrasound, cube tracker and phantom; and (**b**) System in use: Navigating the tracked US probe to the holographic standard plane while wearing the HoloLens 2.

Figure 1 provides an overview of the system.

## 2. State of the Art

The visualisation of the US plane in its physical location was one of the earliest recognised applications of augmented reality, with the navigation of breast needle biopsy having been proposed as far back as 1996 [4]. These concepts have continued and been updated, with preliminary phantom experiments showing the potentially improved performance of biopsy needle placement [5]. Needle placement still dominates the literature in AR ultrasound guidance and training. In common with many AR systems for surgical guidance, such solutions have been proposed for some years, but remain as either lab-based experiments or small clinical studies. The lack of translation to the clinic may be due to a number of factors, including registration accuracy and as well as human factors and perceptual issues such as inattention blindness, where the augmented view obscures the visualisation of the real scene [6].

### 2.1. AR-Assisted Ultrasound Training

Augmented reality obstetric ultrasound training has received relatively limited attention in the literature [7]. We examine some approaches to AR guidance for ultrasound training in other fields. Magee et al. continued the earlier work in needle guidance by developing an augmented reality simulator. Using a simulated ultrasound on a mannequin torso phantom coupled with a mock US probe, they created a training system for needle guidance. A significant user study of 34 consultants and 25 registrars gave a favourable opinion in general, but noted that the haptic feedback was not realistic.

Focusing on a low-cost ultrasound training platform, Shao et al. developed a body pose estimation-based platform for at-home skill development that only requires a printed ArUco marker attached to a simulated probe and a computer with a webcam [8]. They provided a simple system that can be conveniently used by trainees without the need for a real ultrasound machine. A user study demonstrated the utility of this concept, but revealed that the use of pre-recorded US data prevents students from learning US image optimisation. The absence of a real US probe also limits the realism of the training experience.

In an attempt to offer physicians a more accessible US training solution, Costa et al. addressed the problem that conventional simulators require special hardware and developed a HoloLens application that tracks a QR code attached to a Clarius (https://clarius.com/, accessed on 27 December 2022) wireless US probe [9]. Tracked US probe movements are then fed into simulation software that runs on an external computer, which returns an aligned US slice to the HoloLens at the location of the tracked QR code. A laboratory assessment of accuracy and precision showed good results, but there was no user study included.

Simulation systems offer the possibility to practice clinical skills in a controlled environment. Virtual reality training for obstetric US shows some promise and has been proposed for rehearsal before clinical training [10]. Augmented reality can also provide US simulation using video see-through devices [11]. The closest system to ours is the Vimedix TEE/TTE simulator (CAE Healthcare, Montreal) (https://www.caehealthcare.com/ultrasound-simulation/vimedix/, accessed on 27 December 2022) which offers idealised, simulated US slices visualised on 3D models from CT. The system was well received in an initial clinical evaluation [12]. While simulation has shown promise, it has not yet been adopted as a standard part of the obstetric clinical curriculum [7].

Our system differs from the above training systems by using a real US scanner on a phantom coupled with augmented reality visualisation. We feel that this enhances the learning experience beyond idealised virtual simulation since the trainee learns the dexterity of real ultrasound, applying the right pressure while using AR to help with spatial awareness and navigation to the desired planes.

### 2.2. Deep Learning-Based Standard Plane Navigation Methods

Aiming to reduce the inter-operator variability and increase workflow efficiency, researchers have implemented US video-based deep learning based methods to automate standard plane identification. Cai et al. used a convolutional long short-term memory neural network to capture spatio-temporal visual attention information in US videos. The learned visual attention maps are then used to guide standard plane detection for the HC, AC and FL standard planes [13]. Wang et al. focused on the HC and AC standard planes only and developed a VGG network-based video frame classification approach that helps operators navigate to these two standard planes [14]. In a second step, using additional ultrasound probe motion data, they implemented an operator skill classification network.

Aiming to reduce the workload of sonographers and reduce the US examination time, Li et al. focused on automated US scanning processes including navigation to standard planes, performed by robot arms [15] via deep reinforcement learning (RL). A virtual RL agent, represented by a virtual US probe, operates in a 3D reconstructed US volume that depicts a virtual patient. Experimental spine imaging tasks based on previous robot arm-based acquisitions of human patient US scan volumes show promising results, but rigid robotic arms may not lead to effective standard plane acquisitions that comply with human operator standards.

These deep learning approaches may provide the standard plane identification required by training systems such as ours. In its current form, CAL-Tutor requires a medical expert to explicitly place virtual standard planes at the baby anatomy, which eliminates the need for automated standard plane identification.

## 3. Proposed Method

The following software was used to implement the mixed reality software that is displayed in the HoloLens:

- Unity game engine v2020.3.14 (https://unity3d.com/unity/whats-new/2020.3.14, accessed on 27 December 2022);
- Mixed Reality Toolkit (MRTK) v2.7.2.0 (https://github.com/Microsoft/MixedRealityToolkit-Unity/releases, accessed on 27 December 2022);
- HoloLensARToolKit: A Unity-based marker tracking software for the HoloLens2 that uses its front-facing camera [16];
- Aurora electromagnetic tracker (https://www.ndigital.com/electromagnetic-tracking-technology/aurora/, accessed on 27 December 2022).

The 3D models were based on data from laser scans of the US probes and a segmented MRI model of the foetus. The SPACE-Fan US phantom contains a skeletal structure, brain, four-chamber view of the heart, lungs, spleen, kidneys, aorta, UV, UA, and external genitalia. The MRI virtual model does not contain all this anatomical detail, but the overall anatomy of the SPACE-Fan phantom is modelled in correct spatial alignment to the surface. A number of markers (Vitamin E capsules) are used for registration, which is currently manually achieved by the user. HoloLens visualisation exhibits some instability as the user moves around, which has been noted by other authors [17]. This is probably due to the sparse scene reconstruction in the on-board SLAM algorithm within the HoloLens 2. Manual alignment by the user from their given perspective, while prone to human error, may reduce inaccuracies due to perception and head tracking. Our approach allows the trainee to train using a clinical US system, rather than a simulator device with synthetic images, as seen in many high-fidelity simulators. The standard planes (HC, AC, FL) are marked by a trainer in advance using the clinical US system. Figure 1a shows the system consisting of a Voluson US scanner, a SPACE-Fan baby and mother’s abdomen phantom, and a ArUco marker cube that is rigidly attached to the Voluson US probe via a wooden stick.

### 3.1. Design of the Mixed Reality Concept

**Figure 2:**
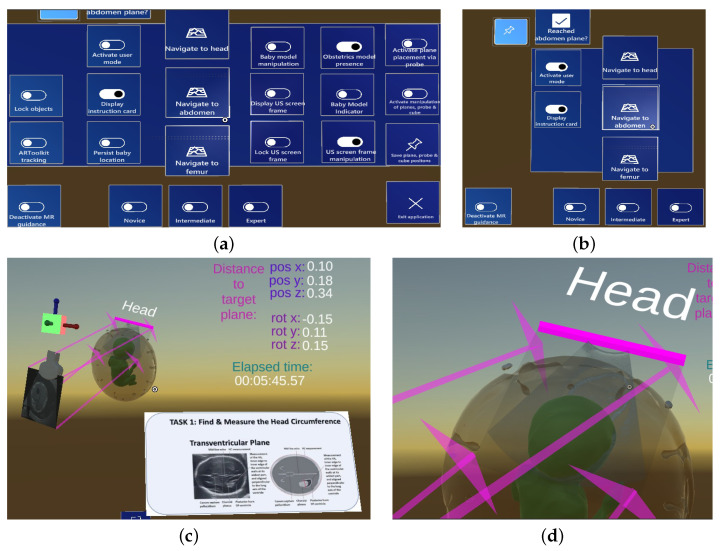
Unity scene showing the various components of the holographic setup: (**a**) complete holographic menu for experts; (**b**) reduced holographic menu for the trainee; (**c**) navigation to head standard plane including the navigation instruction card, navigation arrows, position and rotation offset between probe and target plane and elapsed time for the navigation task; and (**d**) closeup of the head ultrasound plane including the four pink guidance arrows pointing at the plane’s corners.

Our central design consideration for the creation of a mixed-reality ultrasound training approach was an easy-to-use workflow that does not require advanced computer science knowledge. Therefore, all user interaction options are gathered on one holographic menu that shows all available options (without hidden sub menus) and follows the user’s eye gaze but can also be pinned to remain at a fixed 3D position. However, since most of the menu buttons are for experts only, a separate toggle switch buttons disables most of these buttons so that trainees are not distracted by buttons they do not need. Figure 2a,b show the expert and user menu. Figure 2c shows the basic unity components as displayed in the Unity game view. After the 3D reconstructive post-processing of MRI scans, the 3D objects of the baby and obstetric phantom as well as a laser model of the Voluson probe were imported into the Unity scene as .obj files. The objects were scaled to match the size of their physical counterparts. The US probe has an associated plane that represents the US beam produced by the probe. A holographic cube with coordinate axes is rigidly attached to the probe model in a fixed distance, which is used to facilitate a user’s visual confirmation of virtual to real-world alignment when the tracking of the probe is enabled. Figure 2c also shows the navigation components that guide the user to the target standard plane: Four pink guidance arrows originating from the corners of the ultrasound plane that is rigidly attached to the Voluson model point to the matching corners of the standard plane, and thereby serve as an additional visual guidance component that aims to facilitate the visual navigation to the standard planes. A closeup of the pinned head ultrasound plane with guidance arrows can be seen in Figure 2d. In addition to the guidance arrows, the relative distance between two US planes is displayed via position and rotation x, y, z coordinates. The elapsed time, which starts counting after the trainee presses the navigation start button on the holographic menu, serves as an additional aid to make trainees aware of the time it takes them to reach the standard planes during a training session.

#### 3.1.1. Ultrasound Probe Tracking

The tracking of the Voluson ultrasound probe has been realised via a Unity asset named HoloLensARToolkit [16], which is a Universal Windows Platform (UWP) adaption of the well-known ARToolKit open source computer tracking library for augmented reality applications. HoloLensARToolkit accesses the HoloLens’s built-in webcam and tracks printed QR code-based markers. We used the cube00-05-a4 marker which is part of the toolkit’s github project and attached the printed paper cube to the US probe via a wooden stick, as seen in Figure 1b. A holographic counterpart of the QR code marker cube the including x, y, z, the coordinate axes and same relative dimensions is intended to help users visually confirm that the probe is being correctly tracked.

#### 3.1.2. Holographic Guidance during Standard Plane Navigation

Trainees are given several pieces of holographic guidance information designed to help them navigate the US probe to one of the three standard planes, as shown in Figure 2c. This guidance information appears after the trainee has started the navigation phase via holographic button click and comprises the following components:

- Instruction card: The card is a 2D plane with an example image of the standard plane and text explaining how to find the standard plane. The plane can be scaled and positioned anywhere in the scene via the MRTK’s hand gesture-based object interaction.
- Guidance arrows: Four pink arrows emanating from the edges of the US plane attached to the holographic Voluson probe point to the four edges of the standard plane positioned at the respective baby location. The guide arrows are intended to enable the user to navigate to the standard planes more efficiently.
- Numeric offset between the source and target US plane: The relative distance between the US plane attached to the probe and the standard plane is displayed in the upper right corner of the user’s field of view via six numbers: position offset x, y, z and rotation offset x, y, z. These numbers are intended to help trainees verify that the standard plane was positioned in a precise manner.
- Directional indicator: The indicator is a standard MRTK asset consisting of a chevron symbol pointing to the standard plane, helping trainees maintain a broader sense of direction when needed.

### 3.2. User Workflow

The CAL—Tutor application offers three different steps that have to be performed in sequence in order to allow trainees to use its full potential:

#### 3.2.1. Manual Registration of the Baby Model

**Figure 3:**
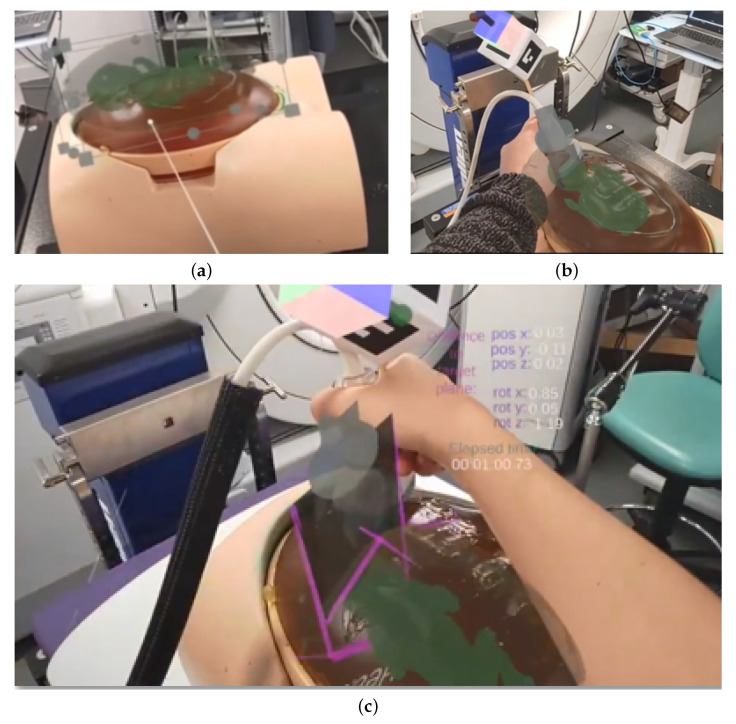
Illustration of the CAL-Tutor’s user work flow phases, shown from the HoloLens 2 perspective: (**a**) The initial manual registration of the baby model (by the expert); (**b**) the manual placement of the holographic standard planes at their respective baby locations (by the expert); and (**c**) trainee navigation to the standard planes.

In the first phase of the application, a medical expert (the trainer) is expected to manually align the holographic baby model to its physical counterpart via hand gesture interaction (Figure 3a). The holographic baby model has been scaled to the actual size of the physical phantom and cannot be rescaled; only translation and rotation is allowed to manually align the model.

As soon as the baby model was manually aligned, the experts confirmed its definitive location via a holographic button click which freezes the model so that it cannot be moved anymore.

#### 3.2.2. Standard Plane Definition

**Figure 4:**
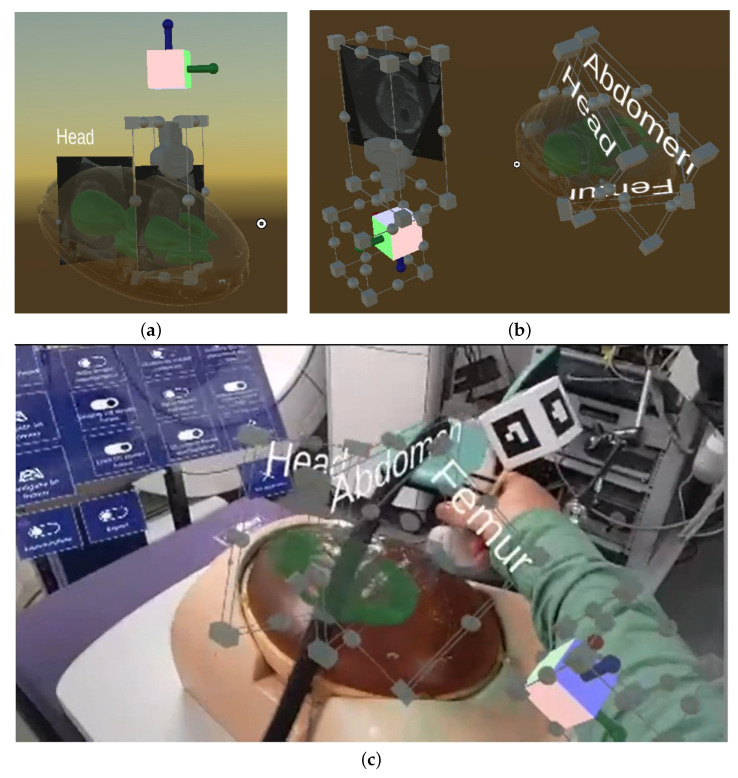
Hologram alignment options: two options of a standard manual plane definition: (**a**) placement of new standard planes via the US probe; (**b**,**c**) adjustment of already existing standard planes whose coordinates have been loaded via a .csv file—(**b**) Unity concept and (**c**) HoloLens 2 view.

After the baby model was manually aligned, the expert was given two options to place the three standard planes HC, AC and FL to their respective anatomical locations of the baby model. The first option is to use the tracked ultrasound probe and place it at the respective standard plane locations with respect to the baby phantom, and place each plane individually by clicking on a holographic button that creates the standard plane by taking a snapshot of the US video that is streamed onto the US plane relative to the probe. Figure 4a shows the concept of placing standard planes via a Unity scene: the ultrasound slice that denotes the head standard plane is placed at the respective anatomical head location and labelled accordingly, while the Voluson probe model is positioned in such a way that the next standard plane (the abdomen) can be placed. In Figure 3b, the manual placement of the head standard plane is shown from the HoloLens 2 perspective.

The second standard plane definition option is to use already existing standard planes positioned in their respective locations in relation to the baby model and whose x, y, z position and rotation coordinates can be loaded via a .csv file. The expert can manually position these planes via hand interaction and save the new coordinates to the .csv file. In addition, the locations of the virtual cube and US probe can be manually adjusted as well as saved in the .csv file. In Figure 4c, a Unity scene is shown that illustrates the concept: the three standard planes as well as the cube and probe have their MRTK-based BoundsControl and ObjectManipulator C# scripts enabled and can be manipulated. In Figure 4b, the manual placement of existing standard planes is shown in a laboratory setup: An expert scans the obstetrics phantom using a Volusion US scanner in order to find the exact locations of the standard planes, and then adjusts the holographic standard planes accordingly via manual interaction.

The holographic model of the US probe has an attached plane that approximates the shape and location of the US beam that is being emitted by the real probe. The virtual plane does not show the live US stream. This visualisation will not be available in clinical practice and we believe that a view of the US plane cut through the baby anatomy, where the user relates this to the ultrasound image on the scanner screen, provides effective training.

After the expert has reached the location of the standard plane, they pin the plane via holographic button click, which creates a clone of the virtual plane. In addition, the cloned US plane contains a pink bar that marks the probe-sided edge of the plane and helps trainees identify from which side they must approach the plane. A text label (‘Head’, ‘Abdomen’, ‘Femur’) helps identify the pinned plane.

After an expert user has placed the standard planes, an unwanted hologram shift could occur when the HoloLens performs a new spatial mapping of the scene, for example, when the expert takes the HoloLens off and puts it back on. In such a case, both the baby model and the standard planes could be shifted, so that a new placement of both the baby model and the planes may be necessary. In order to facilitate a new manual alignment of the holographic content, experts have the option to lock the spatial relationship between the baby model and the pinned standard planes via holographic button click. This way, only a second manual alignment of the baby model is required; the standard planes will remain at the same location relative to the baby anatomy.

#### 3.2.3. Trainee Navigation to Standard Plane

In the third and last phase of the CAL-Tutor application, a trainee navigates the tracked US probe to the location of the previously pinned standard planes (Figure 3c). Since most of the buttons of the holographic menu are only intended to be used by experts and would therefore distract trainees, a separate toggle switch button allows users to switch to an easier menu with fewer buttons (Figure 2a,b).

Each navigation phase starts when a trainee clicks the ‘Navigate to <target anatomy>’ button, for example, ‘Navigate to head’. Holographic guidance information appears in the scene that helps trainees find the standard planes (see Section 3.1.2). During this probe navigation phase, trainees may still look at the physical US screen of the Voluson US system in order to visually confirm that the standard plane was reached.

When a trainee is confident that the standard plane has been reached, they confirm this step via holographic button click, and move on to the next standard plane.

### 3.3. User Data Recording

The HoloLens 2 provides a rich source of information that can be used to gather meaningful user motion data. These motion data can then be further analysed and may lead to new insights about user behaviour. To this end, the CAL-Tutor application records user motion data during the standard plane navigation sequences and stores these data in a separate .csv file that can be downloaded via the HoloLens’ device portal. Currently, we record specific components from the user’s head, hand and eye gaze as well as the US probe motion. The eye gaze data indicate which holographic object the trainee is looking at.

In addition to the HoloLens and ARToolkit data, an external camera records the overall scene, and the US video feed from the Volusion US scanner is recorded as well. Table 1 lists all data that are being recorded.

In order to be able to record when the user is looking at the physical US screen, a holographic frame can be added to the scene and manually aligned to the US screen, so that information about when users are looking at the screen can be recorded.

### 3.4. User Study

In order to investigate the potential benefits of holographic guidance during US probe navigation, a small questionnaire-based user study with six engineering students was conducted to evaluate the users’ personal impression of the CAL-Tutor system’s usability. Two questionnaires had to be filled out after the navigation tasks were completed:

1. NASA Task Load Index (TLX)-based workload assessment via five seven-point scales with 21 graduations (from very low to very high);
2. Product assessment (user experience) via twenty six seven-point scales using different product characteristics.

In addition to the two questionnaires, the participants left personal qualitative notes regarding their experience with the CAL-Tutor system.

Using a real Voluson US scanner and a SPACE-Fan trainer phantom, the CAL-Tutor application was used to guide users to the three standard planes HC, AC and FL. Before the study began, a researcher completed the manual alignment of the holographic baby model to the phantom and placed the three standard planes at their respective locations relative to the holographic baby model. Each participant received instructions on how to use the CAL-Tutor application and how to use the holographic menu to complete the standard plane navigation sequences when wearing the HoloLens 2.

After the introduction and holographic model are set up, the participants are asked to put on the HoloLens device and perform a total of three separate ultrasound (US) navigation tasks to the HC, AC and FL standard planes in this given order twice: once in the baseline condition 1 and once in condition 2, as described in Table 2. The order of conditions was randomised.

## 4. Results

### 4.1. Workload Assessment

**Figure 5:**
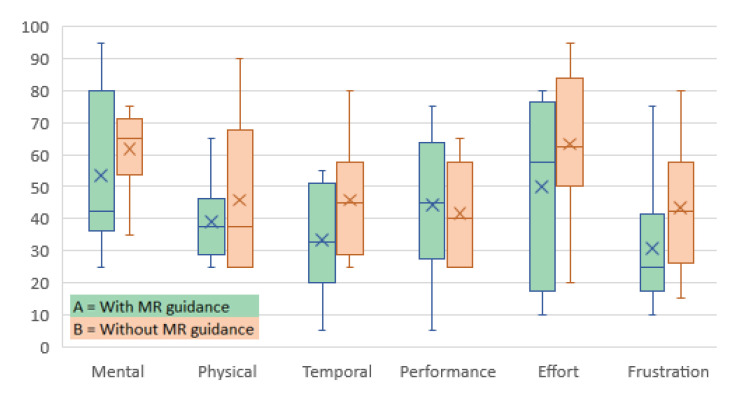
NASA-TLX workload assessment result, represented as a box and whisker chart, grouped into the two experimental conditions A = with MR guidance and B = without MR guidance.

The result of the NASA-TLX workload assessment is shown in Table 3 which presents the individual results for both study conditions A (with mixed reality guidance) and B (without mixed reality guidance), as well as the mean workload values for each workload component. Even though the results are similar for conditions A and B, the mean values are slightly better for condition A, which indicates that mixed reality guidance has a rather positive impact on novice users in terms of finding the three standard planes without prior obstetrics’ ultrasound experience. Figure 5 shows the workload distribution generated from the data depicted in Table 3 which visually confirm the slightly better results of condition A.

### 4.2. Product Assessment

**Figure 6:**
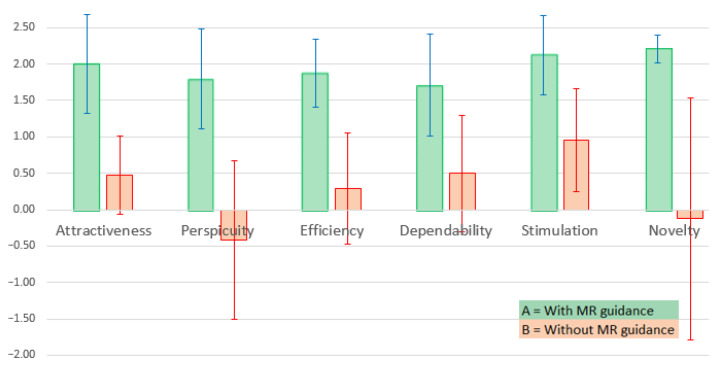
Product assessment result, represented by the comparison of scale means: the chart shows the scale means and corresponding 5% confidence intervals.

In contrast to the workload assessment presented in Section 4.1, the results of the product assessment show a significant difference between conditions A and B, as can be seen in Table 4 and the box whisker plot depicted in Figure 6: the distribution of all assessment categories except “Dependability” is clearly in favour of condition A, meaning that study participants tend to rate the CAL-Tutor system higher when mixed reality guidance was provided.

### 4.3. HoloLens 2 User Motion Data

**Figure 7:**
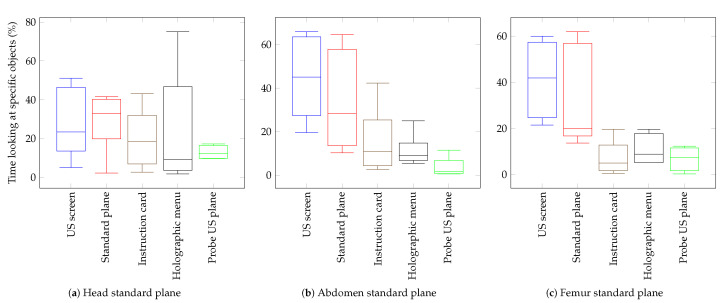
Visual attention profiles of study participants during standard plane navigation: Amount of time (in %) spent looking at specific game objects during navigation to the three standard planes, namely (**a**) head, (**b**) abdomen; and (**c**) femur.

Even though an in—depth evaluation of the gathered HoloLens 2 user motion data is out of the scope of this study, we present a fraction of what could be done to further analyse user behaviour in Figure 7: the visual attention profiles of the study participants for the three mixed reality guided standard plane navigation tasks are presented as box and whisker plots that show the distribution of the time that the users spend looking at specific objects. Despite the small population size, some observations can be made: navigation to the head standard plane appears to require the least amount of time users had to look at the real US screen to find the location of the standard plane (Figure 7a). On the other hand, during navigation to the abdomen and femur standard planes, users spend more time looking at the US screen than looking at the actual holographic standard plane (Figure 7b,c).

In terms of holographic menu interaction, it is worth noticing that the head standard plane shows the highest variation among the three standard plane navigation tasks (Figure 7a) which could indicate that users had to familiarise themselves first with the menu layout before moving on to the subsequent abdomen and femur navigation tasks.

Noting that a higher population size would be required to derive statistically relevant conclusions, this small evaluation of the study participant’s eye gaze data nonetheless suggests that meaningful user behaviour patterns could be derived from such a user motion data collection, which in turn could lead to improved mixed reality experiences.

## 5. Discussion

### Accuracy of Hologram Alignment and Tracking

HoloLensARToolkit provides a simple and convenient tracking method using the front-facing camera of the HoloLens 2 [16]. While this was sufficient to demonstrate the probe tracking, the accuracy of this single camera tracking could be improved. Tracking only works when the user is looking directly at the probe. Furthermore, the processing on the HoloLens leads to some latency and the probe must be moved relatively slowly to maintain tracking. Improved tracking emerged as a common suggestion of our study participants.

Using all the HoloLens sensor cameras in research mode is one option that could improve the range and accuracy of marker tracking. We are also investigating the use of external trackers such as the NDI Aurora electromagnetic tracker. We incorporated the Aurora into our data collection for the assessment and comparison of vision-based tracking. While this could be used as a main tracking device itself, the convenience of visual tracking will have much wider applicability and this is the focus of our research efforts.

The current manual alignment of the obstetrics phantom model to its physical counterpart is labour-intensive, prone to human error and should be automated in future versions. Registration using larger ArUco markers and HoloLensARToolkit tracking gave acceptable results, but some inaccuracies remain. Perceptual inaccuracies using the HoloLens were also noted by many and manual hologram alignment may overcome some of these issues by allowing the user to register the model to their own satisfaction.

## 6. Conclusions

To conclude, we have presented a mixed reality (MR) system that has the potential to improve the learning outcomes for obstetric ultrasound trainees. Initial feedback from six engineers showed that these novice users found that the MR guidance improved many aspects of system interaction, such as efficiency, clarity and stimulation. Our platform also records user motion data, provides interesting insights into user behaviour that could be further analysed, such as distinguishing novice from expert user motion. The software is freely available: https://github.com/manuelbirlo/CAL-Tutor (accessed on 27 December 2022).

## Figures and Tables

**Table 1:** Data recorded by the CAL-Tutor application.

ARToolkit	HoloLens 2	Voluson US scanner
ProbePosition_x, y, z_	EyeGaze: Game object hit Position_x, y, z_	US video
ProbeRotation_x, y, z_	EyeGaze: name of game object user is looking at	
	HandPalmPosition_x, y, z_	NDI Aurora
	HandWristPosition_x, y, z_	ProbePosition_x, y, z_
	HeadPosition_x, y, z_	ProbeRotation_x, y, z_
	HeadRotation_x, y, z_	
External camera		
External camera video of the overall scene		

**Table 2:** The two experimental conditions of the user study.

Experimental Condition
Condition 1 (Baseline): Probe navigation without mixed reality assistance The participant has to wear the HoloLens 2 device during standard plane navigation since user data will be recorded. Despite the fact that the user has to wear the HoloLens 2, no holographic information is being displayed.
Condition 2 (MR guidance): Probe navigation with mixed reality assistance The user is asked to perform the standard plane navigation with holographic guidance which includes the instruction card, the guidance arrows, directional indicator, elapsed time and numerical offset between the probe’s US plane and the target standard plane, as described in Section 3.1.2.

**Table 3:** NASA-TLX questionnaire results for the six study participants (engineering students) in two conditions: With MR guidance (A) and without MR guidance (B).

User Number	Condition	Mental	Physical	Temporal	Performance	Effort	Frustration	Mean
1	A	40	30	50	50	80	20	45
1	B	70	60	80	50	95	80	73
2	A	40	65	5	5	10	10	23
2	B	75	90	40	25	60	30	53
3	A	45	35	25	75	45	30	43
3	B	65	25	50	55	60	50	51
4	A	25	25	30	35	20	20	26
4	B	60	35	50	65	65	45	53
6	A	95	40	55	40	70	75	63
6	B	35	25	30	25	20	40	29
**Workload**	**Value**		**Workload Component**		**With MR guidance**		**Without MR guidance**	
Low	0–9		Mental		53		62	
Medium	10–29		Physical		39		46	
Somewhat high	30–49		Temporal		33		46	
High	50–79		Performance		44		42	
Very high	80–100		Effort		50		63	
			Frustration		31		43	

**Table 4:** Individual product assessment result of all six study participants in two conditions: with MR guidance (A) and Without MR guidance (B).

Scale	Condition	Mean	STD	N	Confidence	Confidence	Interval
Attractiveness	A	2.00	0.85	6	0.68	1.32	2.68
	B	0.47	0.68	6	0.54	−0.07	1.02
Perspicuity	A	1.79	0.86	6	0.69	1.11	2.48
	B	−0.42	1.37	6	1.09	−1.51	0.68
Efficiency	A	1.88	0.59	6	0.47	1.41	2.34
	B	0.29	0.95	6	0.76	−0.47	1.06
Dependability	A	1.71	0.87	6	0.70	1.01	2.41
	B	0.50	1.00	6	0.80	−0.30	1.30
Stimulation	A	2.13	0.68	6	0.55	1.58	2.67
	B	0.96	0.89	6	0.71	0.25	1.67
Novelty	A	2.21	0.25	6	0.20	2.01	2.41
	B	−0.13	2.08	6	1.66	−1.79	1.54

## Data Availability

Not applicable.

## Abbreviations

The following abbreviations are used in this manuscript:

US	Ultrasound
MR	Mixed Reality
HC	Head Circumference
AC	Abdomen Circumference
FL	Femur Length
